# Bacterial communities in natural versus pesticide‐treated *Aphis gossypii* populations in North China

**DOI:** 10.1002/mbo3.652

**Published:** 2018-06-07

**Authors:** Shuai Zhang, Junyu Luo, Li Wang, Lijuan Zhang, Xiangzhen Zhu, Weili Jiang, Jinjie Cui

**Affiliations:** ^1^ State Key Laboratory of Cotton Biology Institute of Cotton Research Chinese Academy of Agricultural Sciences Anyang Henan China

**Keywords:** 16S rDNA, agroecological environment, associated bacteria, azadirachtin, cotton‐melon aphid, *Hamiltonella*

## Abstract

The cotton‐melon aphid, *Aphis gossypii* Glover, is a worldwide‐spreading species, and pesticide‐resistant populations are increasing rapidly. In this study, investigations were performed based on Illumina HiSeq sequencing of the 16S rDNA V4 region for the bacterial communities embodied as intracellular symbionts under natural and in pesticide‐treated populations of *A. gossypii*. The results revealed that more than 82% of bacterial communities belonged to the phylum *Proteobacteria* in which the maximum proportion (53.24%) was of the genus *Arsenophonus; Hamiltonella* composed 22.31; and 1.37% was of the genus *Acinetobacter*. The relative abundance of *Hamiltonella* was obvious, vertically transmitted, divided into two groups, and its infection influenced the bacterial communities in *A. gossypii*. Symbiont density and composition were changed in samples tested on different days. Azadirachtin and phoxim influenced on the composition of bacterial communities. Different biomarkers were used for pesticide‐treated samples with LEfSe results. These findings will increase awareness regarding bacterial communities in naturally occurring populations of *A. gossypii* and pave the way to study the relationship between symbionts and pesticide resistance.

## 1 INTRODUCTION

The cotton‐melon aphid, *Aphis gossypii* Glover, is a species that is increasing worldwide, colonizing more than 600 plant species (Blackman & Eastop, 2000). It causes serious economic losses in agriculture by feeding on plant foliage and spreading viral diseases in plants, such as cotton, cucurbits, and citrus (Thomas, Vanlerberghe‐Masutti, Mistral, Loiseau, & Boissot, 2016). It has been described as holocyclic in North China: eggs hatch in March and after two to three generations, late adults appear and move to cotton fields during late April to mid‐May. Then, its population increases rapidly and becomes the source of crop yield loss from the seedling stage (Wu & Guo, 2003; Xia, 1997).

Cotton‐melon aphid management is primarily dependent on the application of pesticides, such as the use of organophosphates, pyrethroids, and carbamates, but their indiscriminate use caused the development of resistance in its population, which is increasing rapidly (Cao, Zhang, Gao, Liang, & Guo, 2008). To date, the mechanism of pesticide resistance is mostly studied in aphids on the gene level. *A. gossypii* resistance to organophosphates is associated with esterase activity. It has been reported that overexpression of esterases shows higher esterase activity, and expression of mutant carboxylesterases shows lower esterase activity (Field, Blackman, Tyler‐Smith, & Devonshire, 1999; Sun, Zhou, Zhang, & Gao, 2005). Studies have reported that increased metabolic detoxification by increased esterase and oxidase enzymes or modification of voltage‐gated sodium channels decreases the target site sensitivity and induces resistance to pyrethroids (Carletto, Martin, Vanlerberghe‐Masutti, & Brevault, 2010). Similarly, it is stated that resistance mechanisms to neonicotinoid pesticides in aphids are based on enhanced detoxification of cytochrome P450 monooxygenases or reduced affinity of the nicotinic acetylcholine receptor for imidacloprid (Bass et al., 2011).

Including aphids, many insect species harbor endosymbionts known as primary endosymbionts and associated bacteria. The special primary endosymbiont *Buchnera aphidicola* is found in almost all aphid species. *B. aphidico*la is transmitted from mother to offspring with high fidelity and synthesizes essential amino acids and other nutrients that complement the host plant diets. Most aphid‐associated bacteria are thought to be vertically transmitted (Li et al., 2016) and have conditionally beneficial fitness consequences for the host, such as host adaptation (Tsuchida, Koga, & Fukatsu, 2004), increased resistance susceptibility to natural enemies and pathogenic fungi (Oliver, Russell, Moran, & Hunter, 2003; Scarborough, Ferrari, & Godfray, 2005; Vorburger & Rouchet, 2016), and tolerance to high heat shocks (Russell & Moran, 2006). A recent study stated that relative quantities of symbionts have been significantly affected by pesticide resistance (Pan et al., 2013), and gut symbionts enhance pesticide resistance in *Bactrocera dorsalis* (Cheng et al., 2017).

Some studies have been carried out on bacterial communities in *A. gossypii* populations, but bacterial communities and time trends in the natural populations are not clear. In this study, we used the Illumina HiSeq2500 platform, targeting the V4 region of the 16S rDNA to identify the bacterial communities of *A. gossypii* collected from the field at different times and compared them with bacterial communities of *A. gossypii* treated with various pesticides to verify whether the pesticides influence the composition of bacterial communities in *A. gossypii*.

## 2 MATERIALS AND METHODS

### 2.1 Experimental design

The non‐Bt cotton variety CCRI49 was sown in the field at a density of 45,000 hm^2^ in Anyang (36°5′34.8″N, 114°31′47.19″) during April 2016. The plot size for each treatment was kept to 8 × 5 m with three replications.

Five pesticides, including the target and nontarget, were applied against *A. gossypii* on 25 May 2016 when its peak reached to convergence and cotton has one true leaf, while the control treatment consisted of water only (Table 1).

**Table 1:** Pesticide information in this study

Pesticide (concentration)	Formulation	Dosage	Company
Deltamethrin (25 g/L)	EC	450 ml/hm^2^	Bayer Crop Science (China) Co., Ltd
Phoxim (40%)	EC	450 ml/hm^2^	Lianyungang Liben crop science and Technology Co., Ltd
Azadirachtin (100%)	/	90 g/hm^2^	Xi'an Realin Biotechnology Co., Ltd
Imidacloprid (10%)	WP	225 g/hm^2^	Jiangsu KWIN Group
Carbosulfan (20%)	EC	225 ml/hm^2^	Shandong Sino‐Agri United Biotechnology Co., Ltd

EC, emulsifiable concentrate; WP, wettable powder.

Cotton seedlings along with *A. gossypii* were pulled out before pesticide application. Likewise, cotton seedlings along with *A. gossypii* were pulled out 3 days after and 7 days after pesticide application; then, apterous adult aphids were picked up in nuclease‐free Eppendorf tubes for 16S rDNA analysis. Fifty aphids from each were collected, mixed and considered as one sample; one individual per plant was collected to avoid the sampling of offspring from a single mother. To keep the endosymbiotic bacteria community stable, the aphids were picked up immediately after the cotton seedlings were removed, then the tubes with aphid samples were put into liquid nitrogen as soon as possible where they were stored until DNA extraction. The samples were processed one after another. In total, 54 samples were collected and immediately immersed in liquid nitrogen and stored at −80°C for further work.

### 2.2 DNA extraction

Prior to DNA extractions, each aphid sample was washed for 5 min in 70% ethanol and rinsed three times with sterile water in 2.0 ml Eppendorf tubes to remove surface contaminants. TIANamp Genomic DNA Kits (TIANGEN Biotech (Beijing) LTD., China) were used for DNA extractions. The protocol that was followed has been previously reported (Zhao et al., 2016). The quantity and quality of the DNA were measured with a NanoDrop 2000C spectrophotometer (Thermo Scientific, USA).

### 2.3 V4 region of 16S rDNA amplification and sequencing

The V4 region of the 16S rDNA were amplified using the 515F/806R primer; amplicon generation PCR products, quantification and qualification, PCR products mixing and purification, library preparation, and sequencing were done as previously reported (Zhao et al., 2016). Finally, the library was sequenced on an Illumina HiSeq2500 platform and 250 bp paired‐end reads were generated at Novogene Bioinformatics Technology (Beijing, China). Data from 54 samples were obtained independently.

### 2.4 Bioinformatics and statistical analysis

Paired‐end reads were assigned to samples based on their unique barcode and truncated by cutting off the barcode and primer sequence. Paired‐end reads were merged using FLASH (V1.2.7, http://ccb.jhu.edu/software/FLASH/) (Magoc & Salzberg, 2011). Quality filtering on the raw tags and sequence analyses were the same as previously reported (Zhao et al., 2016). Sequences with ≥97% similarity were assigned to the same OTUs. Each OTU was annotated as the taxonomic information used, based on the RDP classifier (Version 2.2, http://sourceforge.net/projects/rdp-classifier/) algorithm according to the GreenGene Database (Desantis et al., 2006; Wang, Garrity, Tiedje, & Cole, 2007).

To account for inequalities in the sequence read depths among the samples, the OTU abundance information was normalized by random selected sequences per sample according to the sequence number of the sample with the least sequences. Subsequent analysis of alpha diversity and beta diversity were performed based on this normalized output data. Alpha diversity analysis included observed species, ACE and Chao1 estimators, Simpson and Shannon diversity indices, and Good's coverage estimates. Linear discriminatory analysis effective size (LEfSe) analysis were performed (Segata et al., 2011). ANOSIM analysis was performed using R software (Version 2.15.3), with a statistical significance threshold of *p* < 0.05. SPSS 20.0 was used for data entry correlation analysis and one‐way ANOVA analysis.

## 3 RESULTS

### 3.1 Sequencing data

The Illumina HiSeq sequencing of the 16S rDNA V4 region amplicons from the natural population of *A. gossypii* yielded 137,579–194,290 raw reads per treatment (three repetitions) (Table 1). The quality filtering sequences were assigned to 264–1,981 OTUs at 97% sequence identity. The rarefaction curves tended to approach the saturation plateau for every sample (Table 2). Good's coverage estimations of sequencing data were above 99% obtained in all samples at a 0.03 dissimilarity cut‐off (Table 2).

**Table 2:** Sequencing analysis of 16S rDNA of *Aphis gossypii* with diversity indices in each treatment

Treatment name	Treatment time (d)	Total reads	Clean reads	OTU number^a^	Observed species	Shannon	Simpson	Chao1	Goods coverage
Control	0	151,802	150,816	310	310	1.22	0.36	481.59	1.00
	3	162,209	161,147	270	206	0.80	0.19	235.00	1.00
	7	165,958	164,615	1,631	1,379	3.48	0.69	1722.73	1.00
Azadirachtin	0	175,807	174,467	1,178	973	1.87	0.40	1214.37	1.00
	3	163,254	162,211	276	206	1.33	0.40	249.33	1.00
	7	151,671	150,444	1,266	1,135	3.49	0.73	1247.22	1.00
Carbosulfan	0	137,579	136,384	1,506	1,349	3.07	0.55	1514.82	1.00
	3	194,290	193,040	478	364	0.98	0.24	506.92	1.00
	7	145,628	144,637	517	460	3.06	0.77	495.13	1.00
Deltamethrin	0	194,026	192,850	400	309	1.19	0.36	444.34	1.00
	3	145,601	144,686	283	224	1.25	0.37	263.76	1.00
	7	155,283	153,899	1,981	1,751	4.03	0.68	1918.29	1.00
Imidacloprid	0	162,818	161,677	1,264	1,060	1.74	0.35	1217.80	1.00
	3	191,471	190,161	434	333	1.84	0.54	400.80	1.00
	7	151,407	150,106	1,040	954	3.95	0.79	1024.14	1.00
Phoxim	0	180,764	179,574	375	296	1.92	0.63	344.12	1.00
	3	172,909	171,790	264	192	0.84	0.21	277.41	1.00
	7	155,166	153,750	1,014	863	2.02	0.42	987.54	1.00

a OTUs (operational taxonomic units) were defined with pairwise 97% sequence identity.

### 3.2 Overview of bacterial communities in *Aphis gossypii*

**Figure 1:**
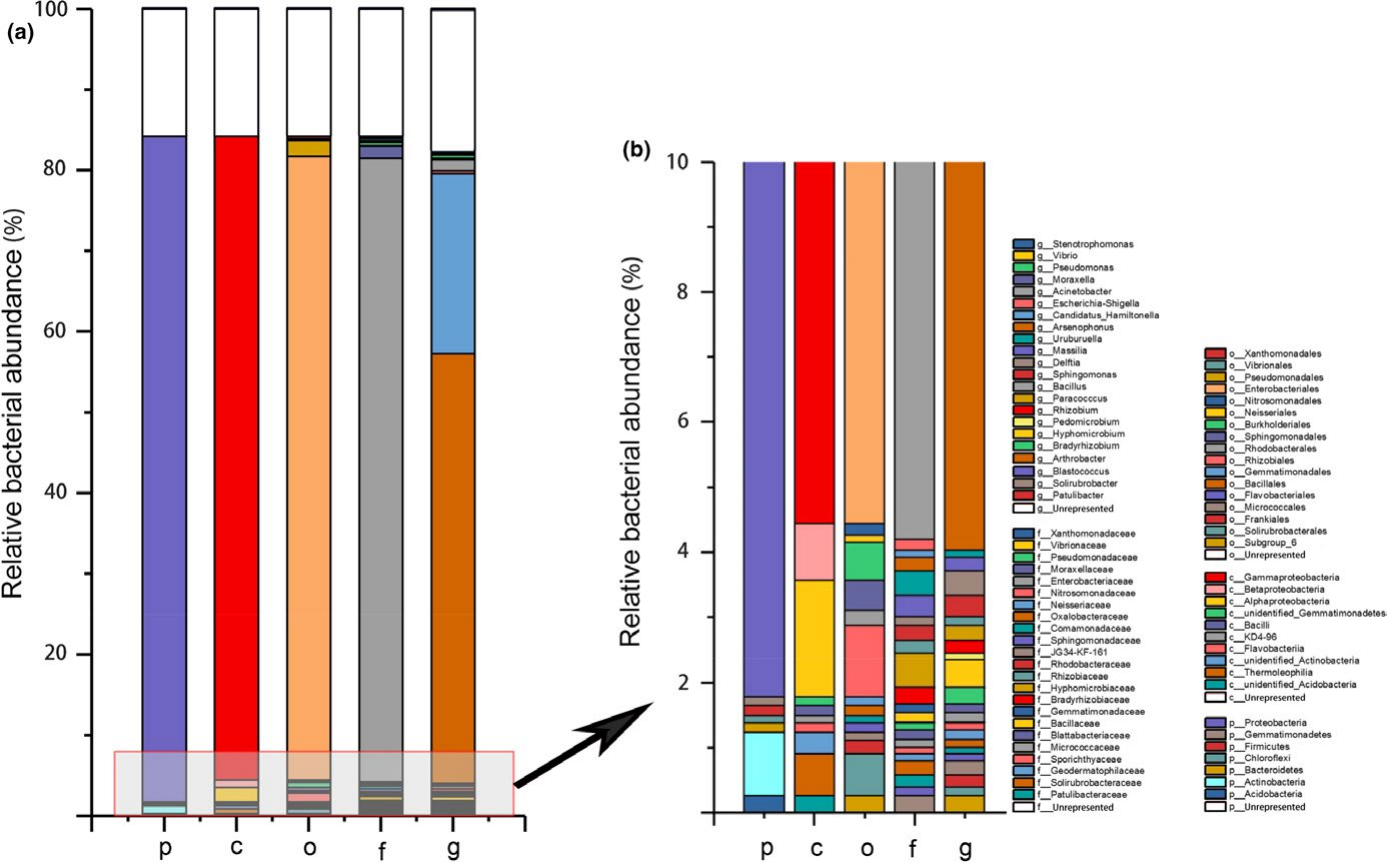
Associated bacteria in *Aphis gossypii* before pesticide application. Average relative abundance of bacterial operational taxonomic units (OTUs) identified across the 18 species before pesticide application on aphid samples at different levels (a) and enlarged part of the low abundance associated bacteria in *A. gossypii* (b). P: phylum‐level; C: class‐level; O: order‐level; F: family‐level; G: genus‐level

The average abundance values across 18 samples of aphid‐associated bacteria were analyzed before applying pesticides. Most bacterial communities were affiliated with the phylum Proteobacteria with a relative abundance of 82.44%. Three aphid‐associated dominant communities from Proteobacteria were Alphaproteobacteria, which remained at 1.79%, Betaproteobacteria at 0.87%, and Gammaproteobacteria at 79.78% (Figure 1). The most abundant associated bacterial families were Enterobacteriaceae, which accounted for 77.29%. In the genus type, there were 22 genera, which represented more than 0.1%. The most abundant genera were three from which the maximum proportion (53.24%) was of the genus *Arsenophonus,* while *Hamiltonella* remained at 22.31 and 1.37% for the genus *Acinetobacter*. The other genera were less than 1.00%. Unlike the associated bacteria, *Buchnera* was in the mycetocyte of aphids, usually known as primary symbionts, and its abundance in *A. gossypii* was 2.92‐fold of the total associated bacterial reads.

**Figure 2:**
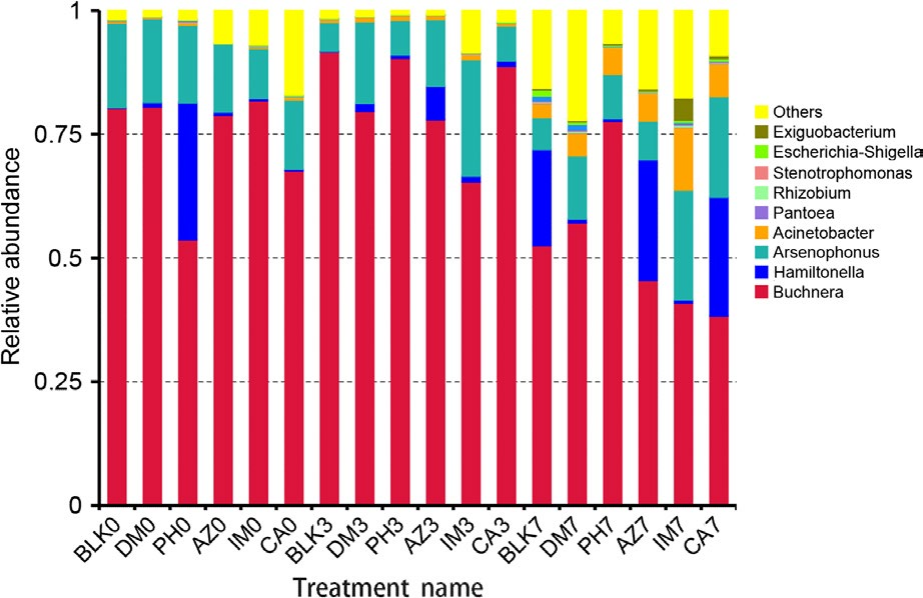
Top 10 most abundant associated bacteria in *A. gossypii* with different pesticides treatment. The abundance values of the OTUs were analyzed at the genus‐level across the pesticides and the control at the genus‐level. BLK0, BLK3, BLK7: used water as a control treatment plot before treatment and after treatment 3 and 7 days; AZ0, AZ3, AZ7: before application of azadirachtin and after application of azadirachtin 3 and 7 days; CA0, CA3, CA7: before application of carbosulfan and after application of carbosulfan 3 and 7 days; DM0, DM3, DM7: before application of deltamethrin and after application of deltamethrin 3 and 7 days; IM0, IM3, IM7: before application of imidacloprid and after application of imidacloprid 3 and 7 days; PH0, PH3, PH7: before application of phoxim and after application of phoxim 3 and 7 days

The abundance values of the top 10 most abundant OTUs were analyzed at the genus‐level across the pesticides and control. Generally, *Arsenophonus*,* Hamiltonella*, and *Acinetobacter* were the most abundant genera of aphid‐associated bacteria. Primary symbionts of *Buchnera* in *A. gossypii* were more abundant than total associated bacterial reads in most treatments. The abundance of *Buchnera* was decreased after insecticide applications except in phoxim‐treated samples, which have more abundant *Hamiltonella* before insecticide applications. Both in the insecticide treatment samples and control sample, the abundance of *Acinetobacter* was increased, but the abundance of *Acinetobacter* was higher in the insecticide treatment samples compared to the control sample, and there were 4.60–6.60% and 2.83%, respectively (Figure 2).

### 3.3 Infection of *Hamiltonella*

**Figure 3:**
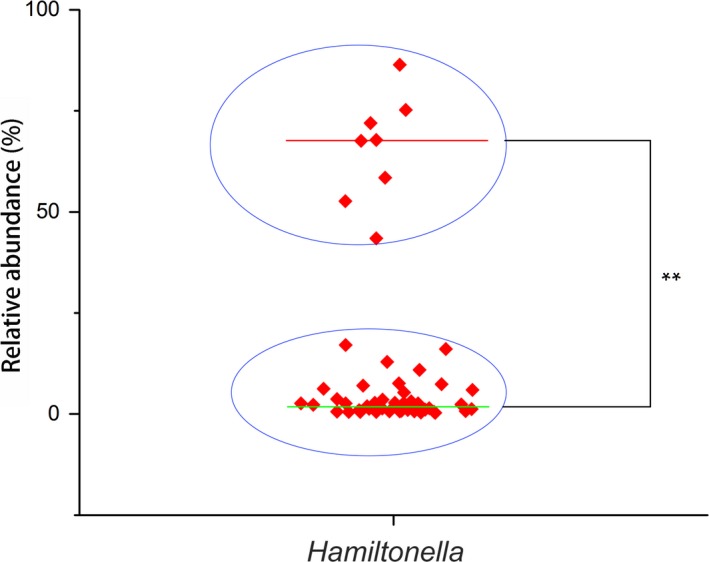
The relative abundance of *Hamiltonella* in *A. gossypii* samples. The 54 aphid populations were divided into two groups (as blue cycle show) by relative abundance of *Hamiltonella*. One group was composed of eight highly abundant populations, and another group has low relative abundance. “**”: one‐way ANOVA analysis use arcsin square root transformed data with SPSS 20.0, *p* < 0.01

The relative abundance of *Hamiltonella* was divided into two groups of 54 samples, with 0.28–86.38% of the associated bacteria. The aphid population was divided into two groups by the relative abundance of *Hamiltonella* (*p* < 0.01). One group composed of eight samples had a high‐relative abundance (43.49–86.38%), and another group has a low‐relative abundance (0.28–17.04%) as shown in Figure 3. The eight high *Hamiltonella*‐infected samples included one sample collected before application of the pesticides, two samples that were collected at 3 days, and five samples that were collected at 7 days. The relative abundance was increased in the samples composed of a relatively high abundance of *Hamiltonella* (*r* = 0.51, *p* = 0.034), but not the samples that contained a relatively low abundance of *Hamiltonella* (*r* = 0.083 *p* = 0.316).

The infection of *Hamiltonella* influenced the primary symbionts in aphids, the relative abundance of *Buchnera*, which were negative according to the relative abundance of *Hamiltonella* (*r* = −0.684, *p* < 0.01). In contrast, the infection of *Hamiltonella* did not influence the main aphid‐associated bacteria, such as *Arsenophonus* (*r* = −0.158, *p* = 0.253), *Acinetobacter* (*r* = −0.044, *p* = 0.749), *Delftia* (*r* = −0.033, *p* = 0.726), *Escherichia‐Shigella* (*r* = −0.034, *p* = 0.807), *Pseudomonas* (*r* = −0.011, *p* = 0.938), *Rhizobium* (*r* = 0.054, *p* = 0.700), *Sphingomonas* (*r* = 0.064, *p* = 0.643), and *Vibrio* (*r* = 0.113, *p* = 0.416).

### 3.4 The time trends of natural bacterial communities

In the plots that were used as controls with no pesticide application, the OTU numbers Shannon, Simpson, and Chao 1 decreased on day 3 and increased on day 7. This means that the most OTU numbers and most symbiont diversity were at day 7. The relative abundance of primary symbiont *Buchnera* remained stable among three sampling times (*r* = −0.489, *p* = 0.219).

At different sampling times, the relative abundance of *Acinetobacter* (*r* = 0.862, *p* = 0.006), *Pseudomonas* (*r* = 0.750, *p* = 0.032), and *Rhizobium* (*r* = 0.785, *p* = 0.021) were increased, whereas the relative abundance of *Arsenophonus* (*r* = 0.795, *p* = 0.018) was decreased, and the relative abundance of *Sphingomonas* (*r* = 0.575, *p* = 0.136) and *Delftia* (*r* = 0.518, *p* = 0.188) remained stable.

### 3.5 The influence of pesticide treatments on bacterial communities in *Aphis gossypii*

Bacterial communities between the naturally occurring aphid population and the pesticide‐treated population were analyzed. Most pesticides indicated no influence on the composition of bacterial communities (ANOSIM, *p* > 0.05). Bacterial communities in aphids were significantly affected by plant‐based pesticides, azadirachtin at 3 days (azadirachtin vs. carbosulfan *p* = 0.015, azadirachtin vs. phoxim *p* = 0.018). Bacterial community composition also varied between carbosulfan and imidacloprid (*p* = 0.041) at 3 days.

**Figure 4:**
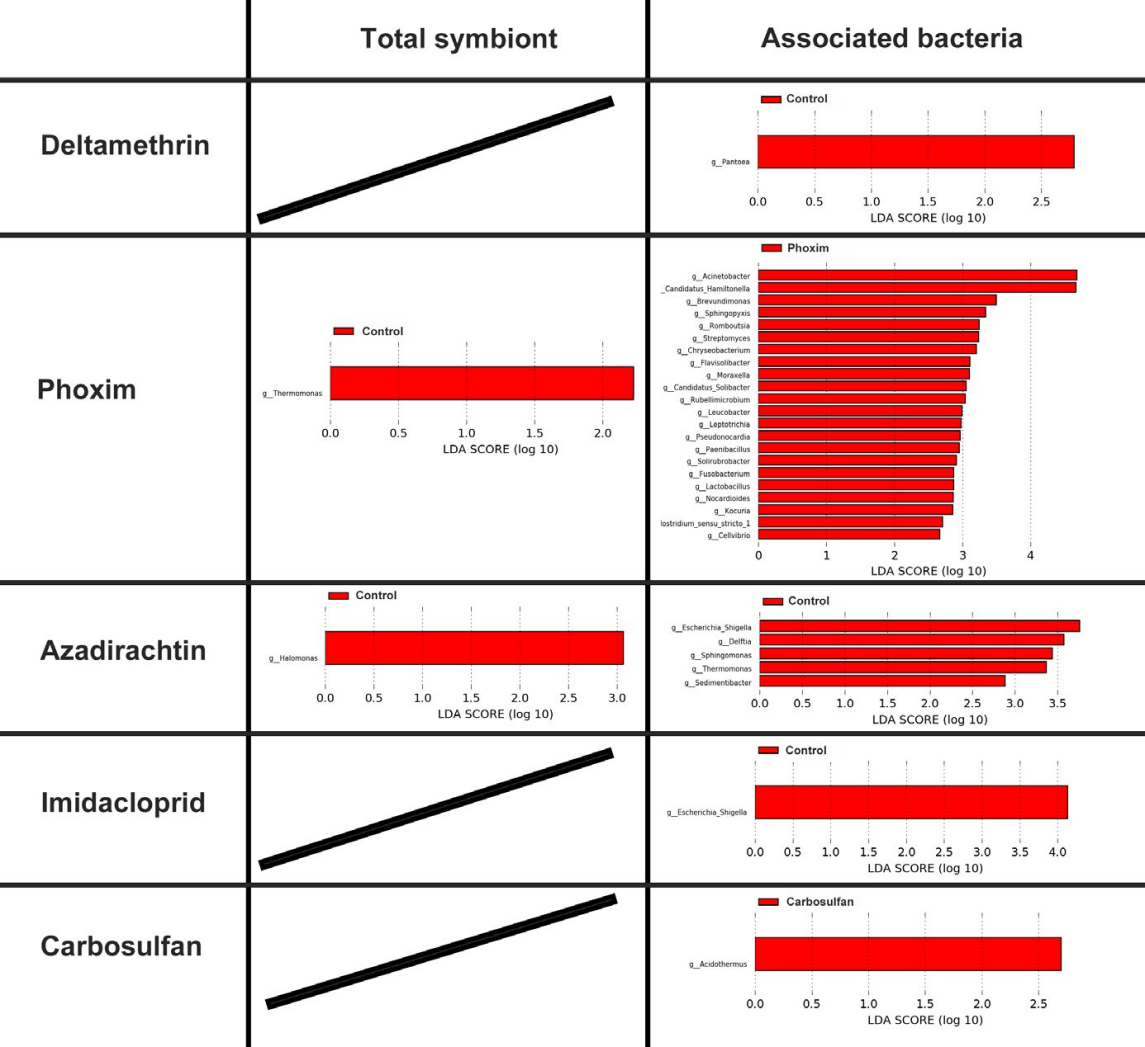
Pesticide treatment effects on bacterial diversity at the genus‐level. Total symbiont: both *Buchnera* primary symbionts and associated bacteria were included in the data analysis. Associated bacteria: only associated bacteria were included in the data analysis. Linear discriminant analysis (LDA) effect size (LEfSe) was used to identify specific phylotypes that were significantly associated with treatments at the genus‐level. An LDA more than two reflects significant differences between the groups. LEfSe analysis provided the list of phylotypes that are differential among dietary supplementations with statistical and biological significance

LEfSe results showed different biomarkers (LDA score > 2.0) in the pesticide‐treated samples and the control samples using the total bacterial communities or only the associated abundant bacterial features. In consideration of the total bacterial communities, biomarker *Halomonas* and *Thermomonas* were from plant‐based pesticides azadirachtin and phoxim‐treated samples, respectively. Thirty biomarkers were identified. When the associated bacteria were considered, there were 22 identified biomarkers that were identified from phoxim‐treated samples 5 days following azadirachtin treatment. Only one was from deltamethrin or imidacloprid or carbosulfan‐treated samples (Figure 4).

## 4 DISCUSSION

Taxonomic classification of insect symbionts revealed that bacterial communities were dominated by only a few phyla, including Proteobacteria, Bacteroidetes, Actinobacteria, and Firmicutes (Jones, Sanchez, & Fierer, 2013). Proteobacteria were the dominant bacterial phylum, including *A. gossypii*, in this study, with the dominant sub‐phylum Gammaproteobacteria. Similar to other insects, the Enterobacteriaceae family constituted the dominant populations in *A. gossypii* (Baumann, 2005; Yuval, Ben‐Ami, Behar, Ben‐Yosef, & Jurkevitch, 2013).

In addition to the *Buchnera* primary symbionts, aphids possess several associated bacteria, which show remarkable differences in morphology, localization, and quantity between lineages and were thought to be polyphyletic (Fukatsu, Tsuchida, Nikoh, & Koga, 2001). The model pea aphid species *Acyrthosiphon pisum* harbors several associated bacteria, including *Serratia symbiotica*,* Hamiltonella defensa*,* Regiella insecticola*, a *Rickettsia* sp., and *Spiroplasma* sp. (Fukatsu et al., 2001; Guay, Boudreault, Michaud, & Cloutier, 2009; Sakurai, Koga, Tsuchida, Meng, & Fukatsu, 2005; Tsuchida, Koga, Fujiwara, & Fukatsu, 2014). In DGGE, 5 bands closely related to *Arsenophonus*,* Pseudomonas*,* Acinetobacter*,* Pelomonas,* and *Burkholderia* were separated from malvaceous and cucurbitaceous populations within Japan and Australia (Najar‐Rodriguez, McGraw, Mensah, Pittman, & Walter, 2009). Eight bacterial groups, including *Arsenophonus*,* Serratia*,* Rickettsia*, and *Wolbachia* were cocolonized on *Colocasia esculenta* and *Alpinia purpurata* across the four Hawaiian Islands (Jones, Bressan, Greenwell, & Fierer, 2011). In this study, the relative abundances of 22 identified genera were more than 0.1%, including the three most abundant bacterial genera *Arsenophonus*,* Hamiltonella* and *Acinetobacter* and the three genera with low abundance (*Pseudomonas*,* Burkholderia*,* Stenotrophomonas*) that have been reported in *A. gossypii* (Jones et al., 2011; Najar‐Rodriguez et al., 2009; Zhao et al., 2016). None of the aphids surveyed were infected with the main endosymbiotic types (*Serratia symbiotica*,* Regiella insecticola*,* Rickettsia*,* Spiroplasma*) that were previously reported for other aphid species (Russell, Latorre, Sabater‐Munoz, Moya, & Moran, 2003).

*Hamiltonella* can be infected with bacteriophage APSEs that reduce the rate of successful parasitism by killing developing wasp larvae (Degnan & Moran, 2008; Laughton, Garcia, Altincicek, Strand, & Gerardo, 2011; Oliver et al., 2003; van der Wilk, Dullemans, Verbeek, & van den Heuvel, 1999). *Hamiltonella* appeared roughly in 14% aphid species (Oliver, Degnan, Burke, & Moran, 2010), it was present in all of our samples and the population was clearly divided into two groups by relative abundance. It indicated that *Hamiltonella* was vertically transmissible in *A. gossypii*, with lower abundance in the *A. gossypii* population; however, it is increasing rapidly in some populations for unknown reasons.

*Hamiltonella* provides strong protection against parasitoid wasps, but appears to have a negative effect on *Aphis fabae* longevity in the absence of parasitoids (Vorburger & Gouskov, 2011). In this study, we found that *Hamiltonella* has a negative effect on the abundance of the primary symbiont *Buchnera* in aphids, but not on the associated bacteria. As *Buchnera* synthesizes essential amino acids and other nutrients for their host aphids, we inferred that *Hamiltonella* has a negative effect on aphids, and reduces the abundance of *Buchnera*.

Environmental factors influenced the symbiont density and composition in normal plots, while both were changed depending on the sampled day. Symbiont density is found to differ significantly between the populations when reared under controlled environmental conditions in mealy bugs (Parkinson, Gobin, & Hughes, 2017). The density of the primary symbiont, *Buchnera*, in pea aphids tends to decrease with host age and increase with rearing temperature (Lu, Chiu, & Kuo, 2014). In this study, bacterial density variation was prospected in the natural adult *A. gossypii* population; the results showed the flexible dynamics of bacterial density over time, although the higher levels of symbionts have no clear benefit to the hosts and therefore appeared to be superfluous (Parkinson et al., 2017).

It was reported that the relative amounts of symbionts were affected significantly by pesticide resistance, and higher *Wolbachia* densities correlated with higher numbers of resistant genes in *Culex pipiens* (Duron et al., 2006). Similarly, high densities of Rickettsia were correlated with higher susceptibility to pesticides in *Bemisia tabaci* (Ghanim & Kontsedalov, 2009). Most studies have focused on the correlation of pesticide resistance to single symbiont densities, and the role of bacterial communities in insect pesticide resistance is still not clear. Because pesticide resistance of *A. gossypii* populations is rapidly increasing, we used low doses of pesticide sprayed on field aphids to study the bacterial communities' dynamics in *A. gossypii*. The results showed that the composition of bacterial communities was stable under four pesticide‐treated samples, but plant‐based pesticide azadirachtin depressed the bacterial communities in *A. gossypii* due to its bacteriostatic activity.

Insecticide applications enriched the insecticide‐degrading bacteria in the agroecosystem (Kikuchi et al., 2012), even though the composition of bacterial communities remained stable under phoxim treatment. There were 22 biomarkers identified at the genus‐level, all of them raised the relative abundance in the phoxim‐treated population. There were some types that are probably involved in insect resistance to pesticides, such as *Acinetobacter*, which was isolated from the midgut of a pesticide‐resistant *H. armigera* (Malhotra et al., 2012), and *Chryseobacterium* isolated from the pyridaben‐resistant population of *Tetranychus urticae* (Yoon et al., 2010). Many pesticides can be degraded by *Kocuria*,* Nocardioides* or *Pseudonocardia* (Bostanian & Akalach, 2006; Chakraborty & Das, 2016; Kumar, Kumar, & Sharma, 2016).

In this study, a broad characterization of the bacterial communities in naturally occurring populations and under pesticide‐treated populations of *A. gossypii* was achieved. Our sequencing data revealed that the relative abundances of 22 genera included more than 0.1% in *A. gossypii* from which *Arsenophonus*,* Hamiltonella* and *Acinetobacter* were the most common bacteria. The symbiont density and composition were changed on different sample days. Interestingly, *Hamiltonella* was obviously divided into two groups according to the relative abundance in *A. gossypii*. It indicated that *Hamiltonella* was vertically transmitted and infection of *Hamiltonella* could influence the bacterial communities in *A. gossypii*. Five pesticides were chosen, including azadirachtin and phoxim, which exhibited influence on the composition of bacterial communities. Different biomarkers were identified in the pesticide‐treated samples with LEfSe results. These findings contribute a new viewpoint to the bacterial communities in aphids, and pesticides can influence bacterial community composition in *A. gossypii* and pave the way to study the relationship between symbionts and pesticide resistance.

## ACKNOWLEDGMENTS

This research was supported by Natural Science Foundation of China (No. 31572015) and National Special Transgenic Project of China (No. 2016ZX08012‐004).

## CONFLICT OF INTEREST

The authors declare that they have no conflict of interest.

## AUTHOR CONTRIBUTIONS

JC and SZ conducted experiments. SZ and LW performed the experiments. SZ, JL, LZ, and XZ analyzed the data. SZ and WJ wrote the manuscript. All the authors reviewed the manuscript.
